# A Case of Medullary Microcarcinoma in the Setting of Cowden’s Syndrome

**DOI:** 10.7759/cureus.26947

**Published:** 2022-07-17

**Authors:** Umberto M Donato, Sebastian A Donato, Kristen Otto

**Affiliations:** 1 Pediatric Oncology, Tampa General Hospital, Tampa, USA; 2 Radiology, Moffitt Cancer Center, Tampa, USA; 3 Pediatric Oncology, University of South Florida Health, Tampa, USA; 4 College of Food, Agricultural and Environmental Sciences, The Ohio State University, Columbus, USA; 5 Otolaryngology - Head and Neck Surgery, Moffitt Cancer Center, Tampa, USA

**Keywords:** hamartomatous, ent procedures, calcitonin, medullary thyroid carcinoma, cowden's

## Abstract

Cowden’s syndrome (CS) is a rare inherited condition characterized by hamartomas in various organs including the thyroid and mucocutaneous tissues as one of the most distinctive features. We present a rare case of Cowden’s syndrome with medullary microcarcinoma of the thyroid, in a 56 year old male with a history of hamartomatous colon polyps, papillomas of the tongue, skin tags, learning disability in the spectrum of autism and macrocephaly. This was evident on immunohistochemical examination of a nodule in the right thyroid lobe. Calcitonin and carcinoembryonic antigen (CEA) positivity along with C-cell hyperplasia were consistent with a medullary microcarcinoma. Total thyroidectomy was performed. Post-operatively margins were uninvolved by carcinoma. Perineural and lymphatic invasion was not identified. Considering the rarity of this condition and the unique presentation of our patient we believe that reporting this case would add more information to the existing fund of knowledge.

## Introduction

Cowden’s syndrome (CS) is a rare genodermatosis inherited in an autosomal dominant manner and is part of a group of disorders involving mutations in the phosphate and tensin homolog (PTEN) tumor suppressor gene. CS is the best described phenotypic presentation of this mutation [1] and is predominantly characterized by multiple hamartomas that can be found in any organ [2]. Typically, patients with CS present with mucocutaneous lesions along with macrocephaly [3]. Cardinal dermatologic features include oral papillomas, acral keratoses, and facial papules. Other common pathological findings associated with Cowden’s are goiter, benign adenomas of the thyroid and hamartomatous polyps of the digestive tract. Even though patients with this disease most commonly present with benign lesions, these patients must undergo long-term monitoring due to their increased risk of developing neoplastic malignancies. Studies have estimated the lifetime risk of thyroid cancer in the setting of Cowden’s syndrome to be 35% [4-6]. The cumulative lifetime risk of any cancer diagnosis in CS patients is 89% [7]. We present a rare case of thyroid medullary microcarcinoma in a 57-year-old male with CS. 

## Case presentation

**Figure 1:**
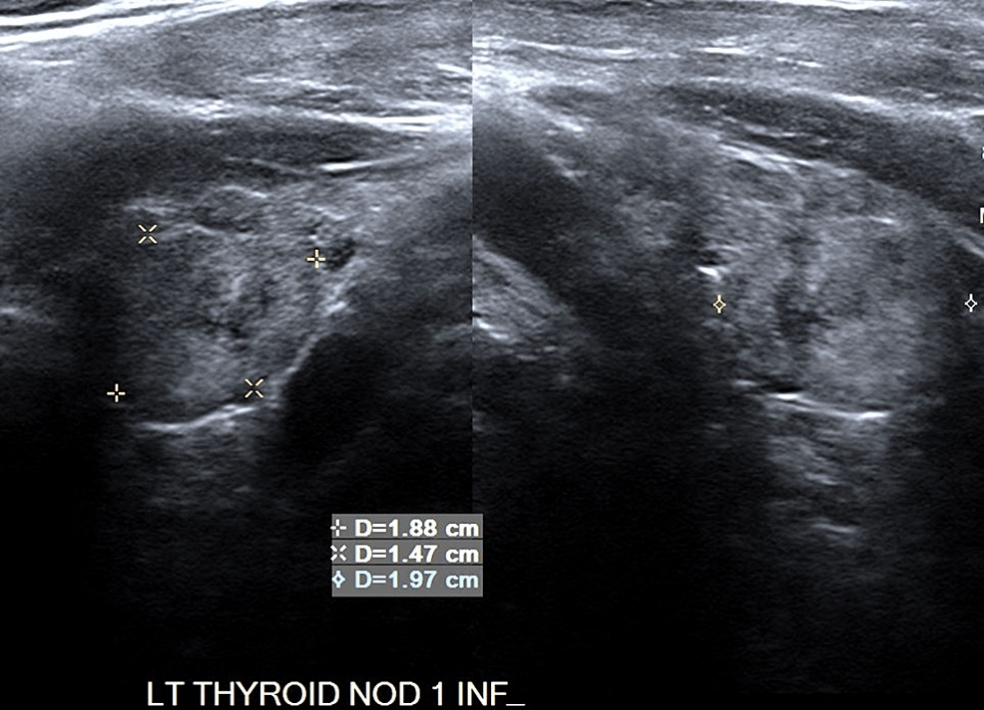
Multinodular Left (Inferior) Thyroid Ultrasound Lesions are marked (x) on image.

**Figure 2:**
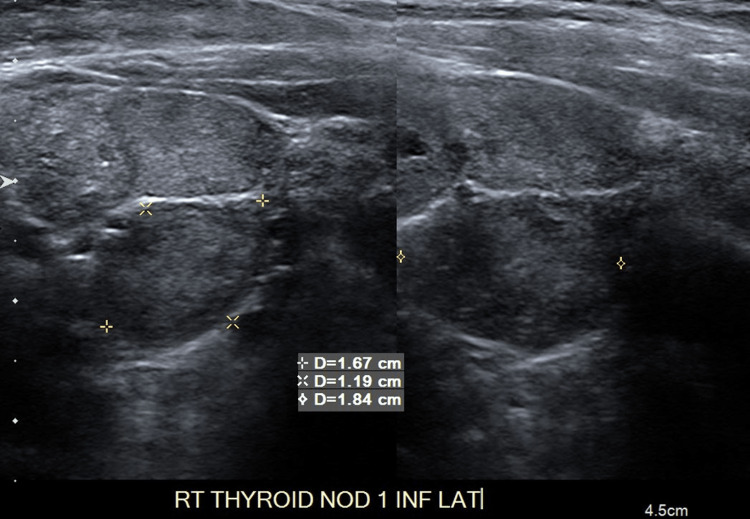
Multinodular Right (Inferolateral) Thyroid Ultrasound Lesions are marked (x) on image.

A 57-year-old man presented at our head and neck clinic for evaluation of thyroid nodules in the setting of Cowden’s syndrome (CS) and consideration for surgery. Latest ultrasound examination showed 11 thyroid nodules with the largest on the right lobe measuring 2.1 cm on the isthmus (Figures 1, 2). These nodules were TI-RADS4, TI-RADS3 and TI-RADS5. Physical examination showed palpable, nontender bilateral thyroid nodules. Lymph nodes in the neck area were unremarkable and respirations were non-labored. The patient also had a history of hamartomatous polyps of the large intestine, hypertensive disorder, kidney cysts, papillomas of the tongue, cutaneous skin tags, biopsy-proven ganglioneuroma and a PTEN likely pathogenic variant (LPV) mutation carrier. He also presented with long-term hypothyroidism treated with levothyroxine. Past neurological and head/face examination showed a learning disability consistent with autism spectrum disorder, obstructive sleep apnea currently treated with continuous-positive-airway-pressure (CPAP) and a relatively enlarged head size. Relevant family history included a mother with breast cancer and a sister with hypothyroidism. Altogether, current symptoms and history were consistent for PTEN gene-related hamartomatous syndrome, specifically CS. Due to elevated risk of cancer associated with CS diagnosis, bilateral thyroid nodularity, and baseline hypothyroidism, the patient was recommended for total thyroidectomy. Two months after the recommendation, the patient underwent a total thyroidectomy and histological analyses were performed on the involved thyroid nodules. These thyroid nodules occupied more than 90% of the parenchyma. These nodules were described as non-invasive follicular thyroid neoplasms with papillary-like nuclear features, measuring 0.6 cm in the greatest dimension and present in the right lower lobe as well. Solid cell nests and lipomatous changes were also noted within the thyroid. Parathyroid gland evidenced no significant histological abnormality. However, the final diagnosis showed a concomitant medullary thyroid carcinoma (MTC) measuring 0.4 cm in the greatest dimension arising in the right thyroid lobe in the background of c-cell hyperplasia and thyroid follicular nodular disease. In order to see whether this MTC occurred via a germline mutation, genetic analysis for a RET protooncogene mutation was performed and results for this test came back negative. This suggested that this MTC emerged spontaneously in the patient. Overall, this patient had two distinct malignancies of the thyroid: a non-invasive follicular thyroid neoplasm with papillary nuclear features (NIFTP) and a "spontaneous" medullary microcarcinoma. These two malignancies rarely occur simultaneously in CS patients, with NIFTPs being more common than medullary microcarcinomas in patients with a CS diagnosis [5]. Immunohistochemical stains were performed with appropriate controls to confirm the presence of the medullary microcarcinoma. The microcarcinoma showed positivity carcinoembryonic antigen (CEA) and calcitonin antibody stains. The thyroid-stimulating hormone (TSH) level for this patient was 1.5 (control reference values, 0.5-5.0) mIU/L, T3 level was 110.0 (control reference values, 80.0-220.0) ng/dL, T4 level was 7.0 (control reference values, 5.0-12.0) ug/dL, and the median calcitonin and CEA levels were 2.0 (control-reference values, 0.0-7.5) pg/mL and 4.1 (control-reference values, 0.0-2.5) ng/mL respectively. Margins were uninvolved by the carcinoma with the closest margin being 1.8 mm from it. The pathological-tumor-node-metastasis (pTNM) classification of this primary unifocal tumor was correspondingly PT1a,N0,M0. 

## Discussion

Cowden’s syndrome is part of the PTEN hamartoma tumor syndrome subgroup which encompasses disorders caused by a mutation on the PTEN gene. Hamartomas are benign tumor-like growths but CS however is characterized by an elevated risk of malignant tumors of the thyroid gland, breast, uterus, and mucocutaneous tissues [8]. The distinctive features of CS include mucocutaneous papules, biopsy-proven trichilemmomas, and oral mucosal papillomatosis. The lifetime risk of cancerous lesions in the breast or thyroid in the setting of CS are estimated to be 25-50% and 35% respectively [4-6,9]. Thyroid disease is one of the most frequent internal manifestations of CS. With regards to thyroid malignancies in CS, follicular and papillary thyroid cancers are the most common types of neoplasms present in patients who underwent total or partial thyroidectomies [10]. Patients may also present with hypo-, hyper-, or euthyroid status. Medullary thyroid carcinomas account for 5-10% of all thyroid cancers and an even lesser percentage in concurrent thyroid disease associated with CS [11]. 

The susceptible gene for CS is10q22-23 identified as a phosphatase and tensin homologue (PTEN) [8]. PTEN is mapped as a tumor suppressor gene responsible for regulating cell cycle progression as well as cell migration and angiogenesis. For our patient, an 84 gene panel test detected a germline likely pathogenic variant of the PTEN gene and was consequently referred to our head and neck clinic to discuss comprehensive surveillance for PTEN gene-related hamartomatous syndrome. 

There is no curative intervention for CS. The principal intervention for the management of CS is mainly rigorous follow-up due to the increased risk these patients have of developing malignant neoplasms across their lifetime. 

Taking into consideration the cardinal pathology of CS, our patient is undoubtedly a unique case. Our patient presented with bilateral thyroid nodules, multinodular goiter, CS, enlarged head size, and long-term hypothyroidism. Due to his history of CS and the bilaterality of his thyroid nodules, the patient was recommended for and later underwent a thyroidectomy. Post-operatively, margins were uninvolved by the carcinoma and the patient's recovery was unremarkable The thyroid nodules were later sent to pathology for immunohistochemical staining. These tests were performed with adequate controls with positive calcitonin and CEA antibody staining fulfilling the major diagnostic criterion of a medullary microcarcinoma in a setting of C-cell hyperplasia [12]. Histological analysis thus evidenced a medullary microcarcinoma 0.4 cm in its greatest dimension located in the right thyroid lobe. Moreover, the absence of a RET protooncogene mutation in the MTC was highly suggestive of a spontaneously occurring/non-germline mutation that led to this neoplasm [13]. This neoplasm occurred in the background of C-cell hyperplasia, thyroid follicular disease and most noticeably CS. A patient diagnosed with CS, a non-germline medullary thyroid microcarcinoma, and concomitant NIFTPs is an exceedingly rare occurrence for which there is hardly any evidence in literature. Without a prior CS diagnosis the importance of rigorous follow-up for cancerous lesions in this patient could have been easily overlooked being that our patient had concurrent medullary microcarcinoma, traits which are not considered pathognomonic for CS. 

## Conclusions

Prompt and accurate recognition of Cowden’s is vital for the early diagnosis of emerging carcinomas associated with this PTEN hamartomatous syndrome. Dermatologists, primary care practitioners, gastroenterologists, and ENTs play a crucial role in identifying the different manifestations of this disease. Creating adequate, multidisciplinary screening plans is essential for the management of CS and its unique presentations. Altogether, this report serves to underscore the presentation, diagnosis and management of a rare case of a medullary microcarcinoma in the setting of Cowden's syndrome,
